# The efficacy and safety of condoliase for lumbar disc herniation: a systematic review and meta-analysis

**DOI:** 10.3389/fphar.2023.1151998

**Published:** 2023-08-21

**Authors:** Zeling Huang, Bo Xu, Yujiang Liu, Hua Chen, Xuefeng Cai, Long Zhang, Xiaofeng Shen, Yuwei Li

**Affiliations:** Suzhou TCM Hospital Affiliated to Nanjing University of Chinese Medicine, Suzhou, Jiangsu, China

**Keywords:** condoliase, lumbar disc herniation, chemonucleolysis, nucleus pulposus, systematic review and meta-analysis

## Abstract

**Background:** Chemonucleolysis is a minimally invasive treatment of lumbar disc herniation (LDH). However, the low specificity of the enzyme and the existence of serious adverse events limit the application of chemonucleolysis. Clinical studies in recent years have shown that Chondroitin sulfate ABC endolyase (condoliase) is a potential therapeutic enzyme for LDH. Aim. A meta-analysis was conducted to determine the efficacy and safety of condoliase in LDH treatment.

**Methods:** We searched Web of Science, Embase, PubMed, and Cochrane Library databases. Two reviewers independently screened articles, extracted data, and assessed the risk of bias. The outcomes were the total effective rate, Oswestry Disability Index (ODI) score change, the proportion of lumbar surgery after condoliase treatment, herniated mass volume change, Pfirrmann grade change, and adverse events. Review Manager 5.3 and Stata 12.0 were used for meta-, sensitivity, and bias analysis.

**Results:** Ten studies were included. A single-arm meta-analysis showed that the total effective rate was 78% [95% confidence interval (CI) 75%–81%], the proportion of surgery was 9% (95% CI 7%–12%), the proportion of Pfirrmann grade change was 43% (95%CI 38%–47%), and the adverse events were 4% (95% CI 2%–6%) after condoliase treatment. The two-arm meta-analysis showed that the ODI score change [standardized mean difference (SMD) −2.46, 95% CI −3.30 to −1.63] and the herniated mass volume change (SMD −16.97, 95% CI −23.92 to −10.03) of the condoliase treatment group were greater than those of the placebo control group, and there was no difference in adverse events between the two groups (OR 1.52, 95% CI 0.60–3.85). The results of sensitivity and publication bias analyses showed that the results were robust.

**Conclusion:** Condoliase intradiscal injection has excellent eutherapeutic and safety for LDH, thus, has considerable potential as a treatment option besides conservative treatment and surgical intervention for LDH.

**Systematic Review Registration:** https://www.crd.york.ac.uk/prospero/display_record.php?ID=CRD42022375492, PROSPERO (CRD42022375492).

## 1 Introduction

Lumbar disc herniation (LDH) refers to the relaxation or rupture of the annulus fibrosus (AF) due to degradation of the intervertebral disc matrix tissue, resulting in extrusion of the nucleus pulposus (NP) from the intervertebral disc (Benzakour et al., 2019). The protruding tissue may irritate or compress the nerve roots, causing symptoms such as lower back pain and/or leg pain, which severely limit the patient’s activity (Samuelly-Leichtag et al., 2022). The occurrence of LDH is related to genetics, excessive loading, and aging (Hoy et al., 2010). Conservative treatment is the primary recommendation for patients with LDH; however, surgery may be considered for patients who fail to respond to long-term conservative treatment. Surgery has advantages over prolonged conservative treatment; nevertheless, it comes with surgery-related risks (Rogerson et al., 2019). New treatment strategies need to be developed to provide options other than conservative treatment and surgical intervention for LDH. Chemonucleolysis involves injecting enzymes into the disc to digest part of the intervertebral disc tissue, reducing the size of the disc herniation and relieving pressure on nerve roots, thereby reducing symptoms (Gentry et al., 1985; Watters et al., 1988). Chemonucleolysis is an intermediate approach between conservative and surgical treatment, and is much less physically and emotionally burdensome than surgery. In 1982, the United States Food and Drug Administration approved chymopapain as a chemonucleolytic drug; however, chymopapain was discontinued in 1999 due to its low substrate specificity, disturbing nerve roots, and anaphylactic reactions (Nordby et al., 1993).

The chondroitin sulfate ABC endolyase (condoliase) is a mucopolysaccharide enzyme. It is highly substrate-specific for chondroitin sulfate and hyaluronic acid (Fan et al., 2022). Therefore, condoliase can specifically degrade proteoglycan-rich NP tissues, whereas the surrounding tissue remains largely unaffected (Ishibashi et al., 2019). The drug regulatory authority in Japan approved condoliase for the treatment of LDH in 2018, and multiple clinical trials have shown its safety and efficacy (Chiba et al., 2018; Matsuyama et al., 2018; Nakajima et al., 2020; Hirai et al., 2021; Inoue et al., 2021; Okada et al., 2021; Banno et al., 2022; Kobayashi et al., 2022; Oshita et al., 2022). Intradiscal injection of condoliase may help patients to return to society sooner. However, some studies have suggested that condoliase has limited clinical efficacy in treating LDH (Ishibashi et al., 2020). Currently, there are no systematic reviews of condoliase for the treatment of LDH, which leaves clinicians with little reference when making decisions. Therefore, we conducted a meta-analysis of the efficacy and safety of condoliase in LDH treatment.

## 2 Materials and methods

The systematic review was registered in PROSPERO (CRD42022375492) https://www.crd.york.ac.uk/prospero/display_record.php?ID=CRD42022375492.

### 2.1 Search strategy

Two reviewers (ZH and BX) conducted an independent literature search. Electronic database search was conducted in PubMed, Web of Science, Embase, and Cochrane Library until October 2022. Keywords used for the search were as follows: “chondroitin ABC lyase”, “condoliase”, “chondroitin sulfate ABC endolyase”, “chondroitinase ABC”, “intradiscal”, “back pain”, “lumbar disc herniation”, and “discogenic”. Using PubMed as an example, the search strategy is as follows:

(1) “chondroitin ABC lyase” [ti, ab] OR “condoliase” [ti, ab] OR “chondroitin sulfate ABC endolyase” [ti, ab] OR “cchondroitinase ABC” [ti, ab]
(2) “intradiscal” [ti, ab] OR ‘back pain’ [ti, ab] OR ‘lumbar disc herniation’ [ti, ab] OR ‘discogenic’ [ti, ab]
(3) (1) and (2)

### 2.2 Inclusion and exclusion criteria

#### 2.2.1 Inclusion criteria

1) Participants: Patients were clearly diagnosed with LDH. 2) Intervention: Intradiscal injection of condoliase. 3) Comparison: Placebo procedure, or none. 4) Study Design: Prospective studies, retrospective studies, and Randomised controlled trials (RCT). 5) Outcomes: The primary outcome was the total effective rate (proportion of individuals with ≥50% pain improvement on the visual analogue scale or numeric rating scale). Secondary outcomes included Oswestry Disability Index (ODI) score change, proportion of lumbar surgery after intradiscal injection of condoliase, herniated mass volume change, Pfirrmann grade change, and adverse events.

#### 2.2.2 Exclusion Criteria

1) Repeated publications. 2) Lack of research on available data. 3) Full-text literature was not available.

### 2.3 Literature screening and data extraction

The literature was independently screened by two reviewers (ZH and BX). The basic information, study design, outcomes, and other data were extracted by two reviewers (ZH and BX). Any inconsistencies were scrutinised by a third reviewer (YL).

### 2.4 Quality assessment of the included studies

Two reviewers (BX and YL) assessed the risk of bias in RCT using to the bias risk assessment tool recommended in the Cochrane Manual (Hopp, 2015). The improved Newcastle-Ottawa scale (NOS) was used to evaluate the quality of non-RCT studies. Based on the original NOS scale, two questions related to the selection and comparison of non-exposed patients and one question related to the evaluation results were reduced, and the question of whether there was financial sponsorship from pharmaceutical enterprises was added (Stang, 2010; Huang et al., 2022). Any inconsistencies were scrutinised by a third reviewer (XS).

### 2.5 Statistical analysis

Review Manager 5.3. was used to analyse the clinical data. For the two-arm study with a control group, count data were evaluated using relative risk (RR) and 95% confidence interval (CI), and measurement data were analysed using the standardized mean difference (SMD) and 95%CI (Huang et al., 2022). For the single-arm study without a control group, the risk difference (RD, %) and 95%CI were used to analyse the event rate (Wang et al., 2021). If *I* ^*2*^ < 50% or *p* > 0.05, the heterogeneity among the included studies was considered small, and the fixed effect model was adopted; Otherwise, the random effects model was adopted (Cordero et al., 2021). Stata 12.0 was used for sensitivity and bias analysis to assess publication bias.

## 3 Results

### 3.1 Search results

**FIGURE 1:**
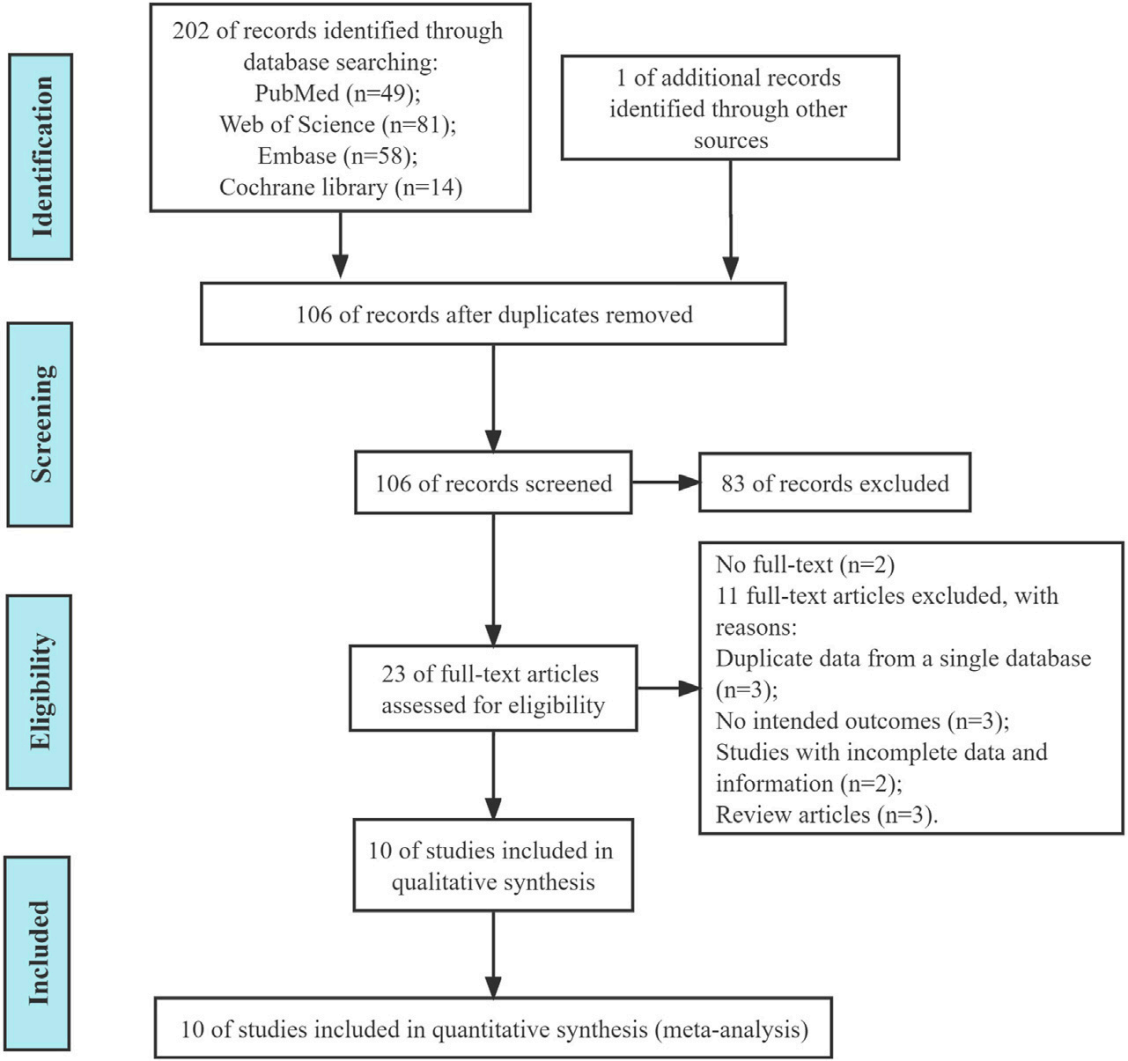
PRISMA flow diagram of the included studies.

We initially retrieved 202 (Figure 1) and deleted 96 duplicate articles. A total of 10 articles were included in this study through screening (Chiba et al., 2018; Matsuyama et al., 2018; Ishibashi et al., 2020; Nakajima et al., 2020; Hirai et al., 2021; Inoue et al., 2021; Okada et al., 2021; Banno et al., 2022; Kobayashi et al., 2022; Oshita et al., 2022).

### 3.2 Characteristics of the included studies

The included studies included two RCT (Chiba et al., 2018; Matsuyama et al., 2018), two prospective (Inoue et al., 2021; Banno et al., 2022), and six retrospective studies (Ishibashi et al., 2020; Nakajima et al., 2020; Hirai et al., 2021; Okada et al., 2021; Kobayashi et al., 2022; Oshita et al., 2022). A placebo was used as a control group in both RCT studies. A total of 259 patients were enrolled in the two RCTS and the remaining eight studies were single-arm trials involving 552 patients. (Table 1).

**TABLE 1:** Characteristics of the included studies.

Author, year	Study design	Sample size	Age (mean ± SD)	Gender (male/famale)	Herniation type	Intervention	Time of assessment (months)	Outcomes
Banno et al., 2022	Prospective study	60	44.5 ± 18.9	37/23	Excluded transligamentous herniation	Condoliase, 1.25U/mL, 1mL, intradiscal injection	12	①③⑤⑥
Chiba et al., 2018	RCT	82/81	TG: 39.5 ± 11.1	TG: 51/31	Excluded transligamentous herniation, or sequestration herniation	TG: Condoliase, 1.25U/mL, 1mL, intradiscal injection	3、12	①②③④⑤⑥
			CG: 39.2 ± 12.4	CG: 51/30		CG: Placebo		
Hirai et al., 2021	Retrospective study	52	45.0 ± 17.7	35/17	Subligamentous herniation, and transligamentous herniation	Condoliase, 1.25U/mL, 1mL, intradiscal injection	6	①③⑤⑥
Inoue et al., 2021	Prospective study	84	44.2 ± 17.1	52/32	Subligamentous herniation	Condoliase, intradiscal injection	6	①③⑤⑥
Kobayashi et al., 2022	Retrospective study	127	46.6 ± 17.1	88/39	Subligamentous herniation, and transligamentous herniation	Condoliase, 1.25U/mL, 1mL, intradiscal injection	3	①③⑤⑥
Matsuyama et al., 2018	RCT	49/47	TG: 41.9 ± 10.9	TG: 38/11	/	TG: Condoliase, 1.25U/mL, 1mL, intradiscal injection	3、12	②④⑥
			CG: 34.0 ± 10.2	CG: 31/16		CG: Placebo		
Nakajima et al., 2020	Retrospective study	42	46.0 ± 13.8	29/13	Subligamentous herniation, and transligamentous herniation	Condoliase, 1.25U/mL, 1 mL, intradiscal injection	3	①⑥
Okada et al., 2021	Retrospective study	82	47.2 ± 15.5	55/27	/	Condoliase, 1.25U/mL, 1 mL, intradiscal injection	6	①③⑤⑥
Oshita et al., 2022	Retrospective study	71	/	38/33	Protruding, subligamentous herniation, and transligamentous herniation	Condoliase, 1.25U/mL, 1 mL, intradiscal injection	3	①⑥
Ishibashi et al., 2020	Retrospective study	34	32.4 ± 13.0	10/24	Subligamentous herniation, and transligamentous herniation	Condoliase, 1.25U/mL, 1 mL, intradiscal injection	3	①③⑥

Abbreviations: TG: treatment group; CG: control group; /: not mentioned; ①: total Effective Rate; ②: ODI, score change; ③: the proportion of operation after condoliase treatment; ④: erniated mass volume change; ⑤: Pfirrmann grade change; ⑥: adverse even.

### 3.3 Quality assessment

**FIGURE 2:**
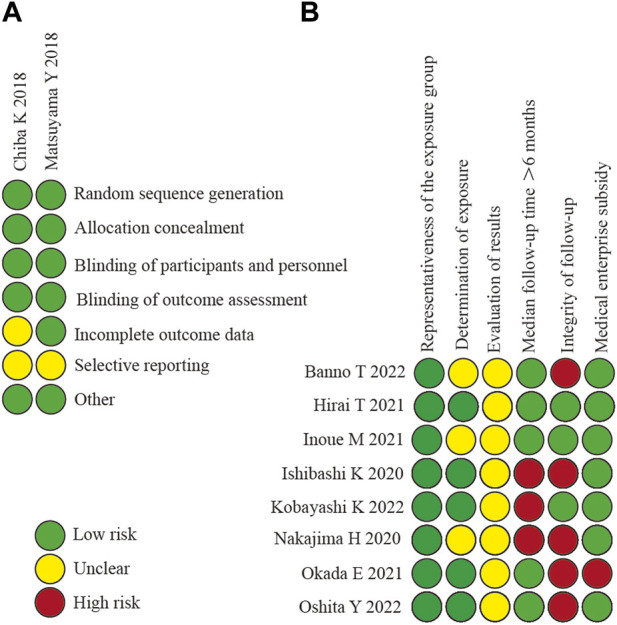
Risk of bias assessment **(A)**: the risk of bias in RCT; **(B)** the risk of bias in non-RCT).

The methodological quality and bias risk of the included RCT and non-RCT studies are shown in Figure 2. The included 10 studies (Chiba et al., 2018; Matsuyama et al., 2018; Ishibashi et al., 2020; Nakajima et al., 2020; Hirai et al., 2021; Inoue et al., 2021; Okada et al., 2021; Banno et al., 2022; Kobayashi et al., 2022; Oshita et al., 2022) were of good methodological quality, and none warranted exclusion in terms of methodological quality.

### 3.4 Results of meta-analysis

#### 3.4.1 Total effective rate

**FIGURE 3:**
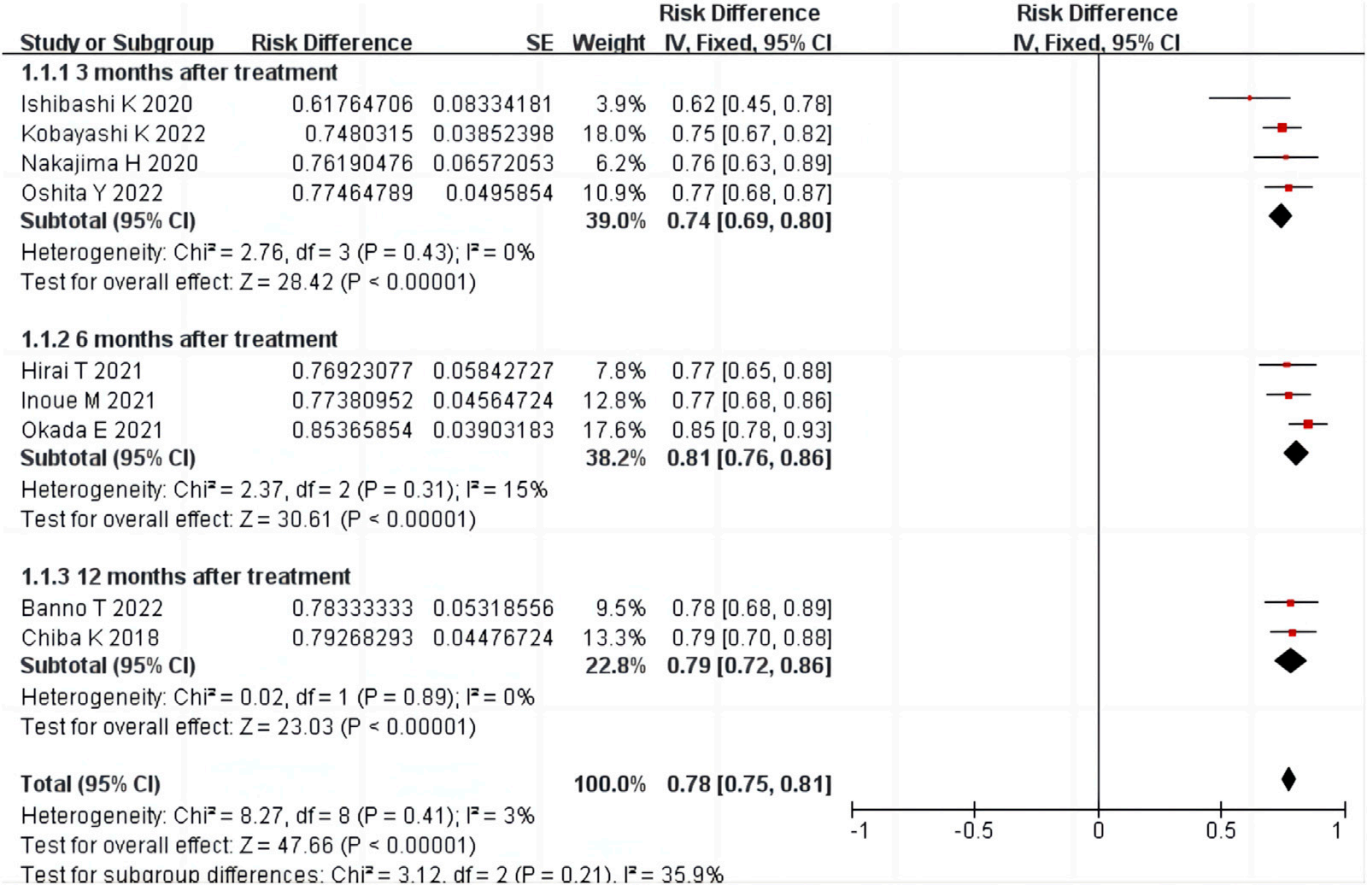
Forest plot of total effective rate.

Nine studies (Chiba et al., 2018; Ishibashi et al., 2020; Nakajima et al., 2020; Hirai et al., 2021; Inoue et al., 2021; Okada et al., 2021; Banno et al., 2022; Kobayashi et al., 2022; Oshita et al., 2022) reported the total effective treatment rate, including 634 patients. There was homogeneity among the studies (*p* = 0.41, *I* ^*2*^ = 3%). The results of single-arm meta-analysis of fixed effects model showed that the total effective rate of condoliase treatment was 78% (95%CI 75%–81%). Four (Ishibashi et al., 2020; Nakajima et al., 2020; Kobayashi et al., 2022; Oshita et al., 2022), three (Hirai et al., 2021; Inoue et al., 2021; Okada et al., 2021), and two (Chiba et al., 2018; Banno et al., 2022) studies were followed up three, six, and 12 months after treatment, respectively. Subgroup analysis was performed according to the follow-up time, and all subgroups were homogeneous (all *p* > 0.05, all *I* ^*2*^ < 50%). Subgroup analysis showed that the total effective rate at three, six, and 12 months after condoliase treatment was 74% (95%CI 69%–80%), 81% (95%CI 76%–86%), and 79% (95%CI 72%–86%), respectively (Figure 3). The results of the RCT by Chiba et al. (2018) showed a higher response rate 12 months after treatment in the condoliase (79.3%) than in the placebo control group (63%) (*p* = 0.02).

#### 3.4.2 ODI score change

**FIGURE 4:**
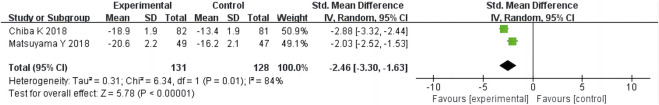
Forest plot of ODI score change.

Two RCT (Chiba et al., 2018; Matsuyama et al., 2018) reported ODI score change in 259 patients. There was a large heterogeneity between the two groups (*p* = 0.01, *I* ^*2*^ = 84%). The results of two-arm meta-analysis of random effects model showed that the ODI score change of the condoliase treatment group was greater than that of the placebo control group (SMD -2.46, 95%CI -3.30 to −1.63) (Figure 4).

#### 3.4.3 The proportion of surgery after condoliase treatment

**FIGURE 5:**
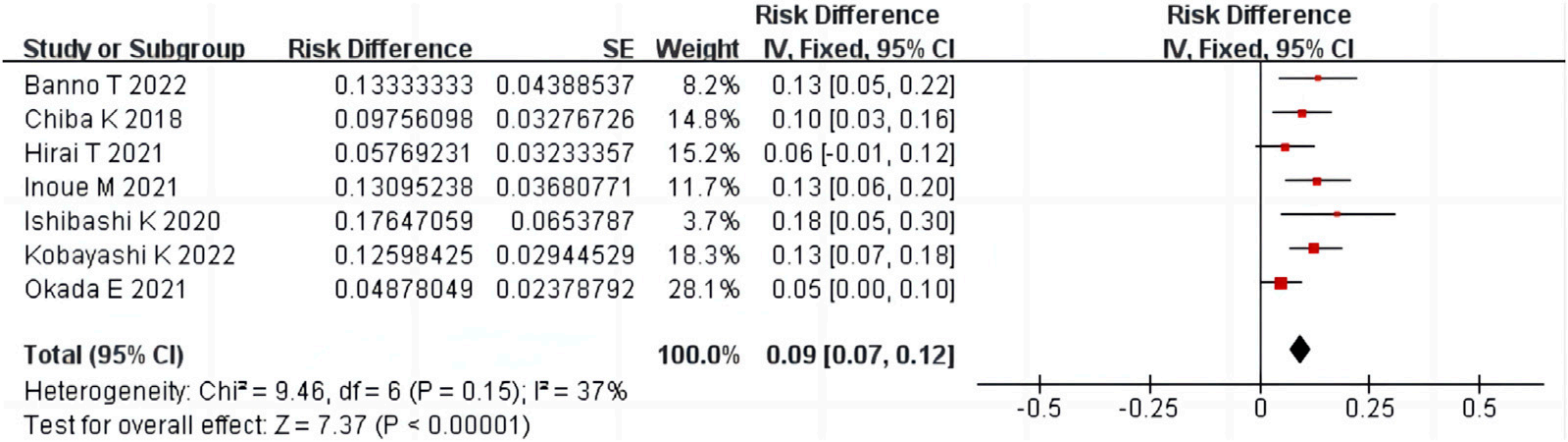
Forest plot of the proportion of surgery after condoliase treatment.

Seven studies (Chiba et al., 2018; Ishibashi et al., 2020; Hirai et al., 2021; Inoue et al., 2021; Okada et al., 2021; Banno et al., 2022; Kobayashi et al., 2022) reported the proportion of surgery performed after condoliase treatment, including 568 patients. There was homogeneity among the studies (*p* = 0.15, *I* ^*2*^ = 37%). The results of single-arm meta-analysis of fixed effects model showed that the proportion of surgery after condoliase treatment was 9% (95%CI 7%–12%) (Figure 5).

#### 3.4.4 Herniated mass volume change

**FIGURE 6:**
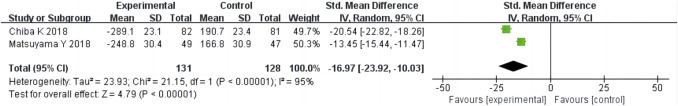
Forest plot of herniated mass volume change.

Two RCT studies (Chiba et al., 2018; Matsuyama et al., 2018) reported a herniated mass volume change in 259 patients. There was a large heterogeneity between the two groups (*p* < 0.001, *I* ^*2*^ = 95%). The results of two-arm meta-analysis of random effects model showed that the herniated mass volume change of the condoliase treatment group was greater than that of the placebo control group (SMD -16.97, 95%CI -23.92 to −10.03) (Figure 6).

#### 3.4.5 Pfirrmann grade change

**FIGURE 7:**
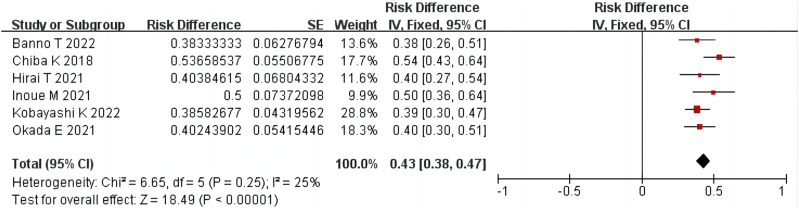
Forest plot of Pfirrmann grade change.

Six studies (Chiba et al., 2018; Hirai et al., 2021; Inoue et al., 2021; Okada et al., 2021; Banno et al., 2022; Kobayashi et al., 2022) including 449 patients reported Pfirrmann grade changes after condoliase treatment. There was homogeneity among the studies (*p* = 0.25, *I* ^*2*^ = 25%). The results of single-arm meta-analysis of fixed effects model showed that the proportion of Pfirrmann grade change after condoliase treatment was 43% (95%CI 38%–47%) (Figure 7).

#### 3.4.6 Adverse events

**FIGURE 8:**
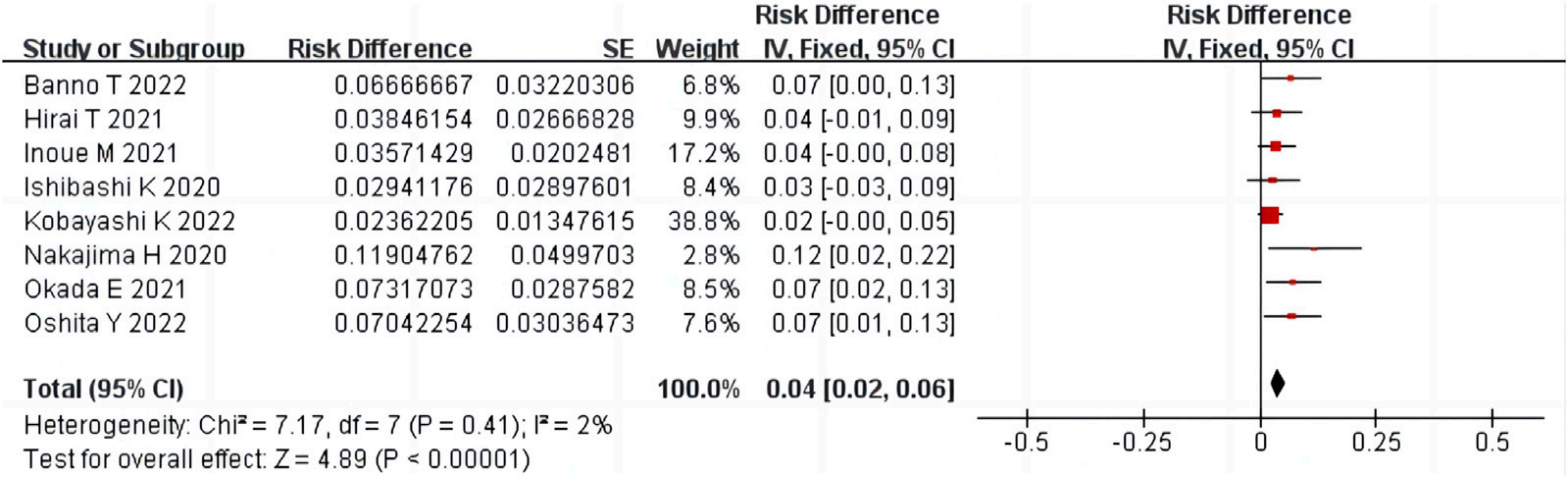
Forest plot of single-arm meta-analysis of adverse events.

**FIGURE 9:**
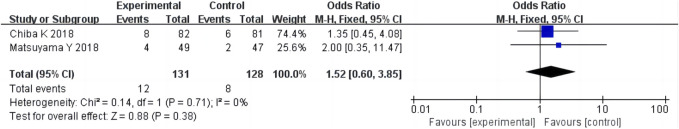
Forest plot of two-arm meta-analysis of adverse events.

Ten studies (Chiba et al., 2018; Matsuyama et al., 2018; Ishibashi et al., 2020; Nakajima et al., 2020; Hirai et al., 2021; Inoue et al., 2021; Okada et al., 2021; Banno et al., 2022; Kobayashi et al., 2022; Oshita et al., 2022) reported adverse events in 811 patients. There was homogeneity among eight non-RCT studies (*p* = 0.41, *I* ^*2*^ = 2%) (Ishibashi et al., 2020; Nakajima et al., 2020; Hirai et al., 2021; Inoue et al., 2021; Okada et al., 2021; Banno et al., 2022; Kobayashi et al., 2022; Oshita et al., 2022). The results of single-arm meta-analysis of fixed effects model showed that the adverse events after condoliase treatment were 4% (95%CI 2%–6%) (Figure 8). There was homogeneity among the two RCT studies (*p* = 0.71, *I* ^*2*^ = 0%) (Chiba et al., 2018; Matsuyama et al., 2018). The results of two-arm meta-analysis of fixed effects model showed the adverse events were the same between the condoliase treatment and placebo control groups (OR 1.52, 95%CI 0.60–3.85) (Figure 9).

### 3.5 Sensitivity analysis and publication bias

**FIGURE 10:**
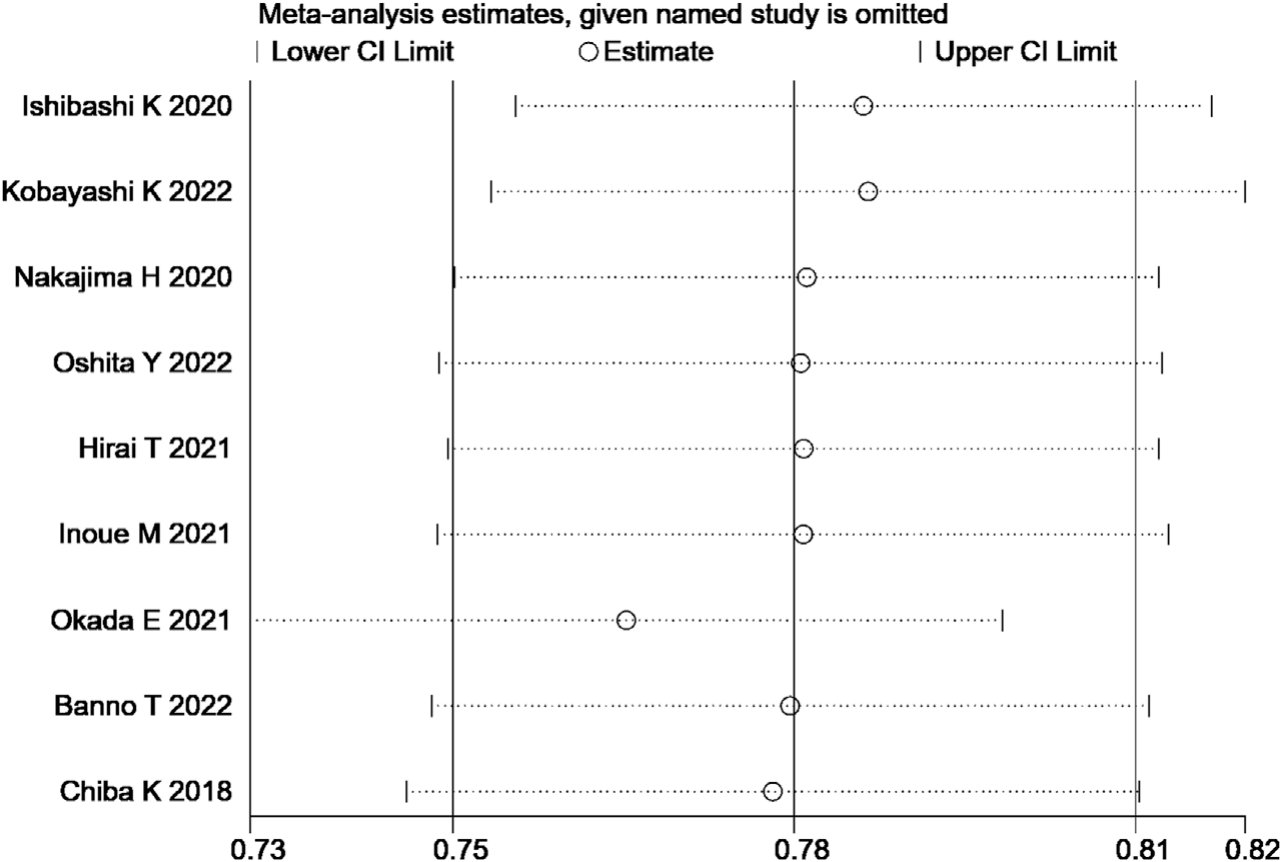
Sensitivity analysis to the total effective rate.

**FIGURE 11:**
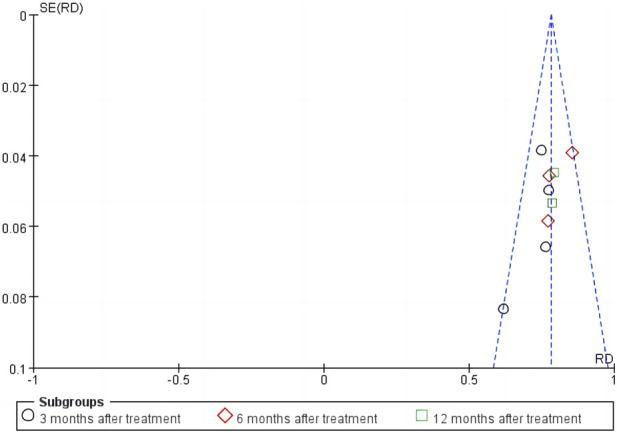
Publication bias of the funnel plot of total effective rate.

The sensitivity analysis of the total effective rate suggested that the meta-analysis results were stable (Figure 10). Analysis of the funnel plot of the total effective rate showed that each study had a symmetric distribution. The *p*-value of the Egger’s test was 0.052, suggesting a small possibility of publication bias (Figure 11).

## 4 Discussion

Chemonucleolysis is a minimally invasive treatment for LDH that does not require general anaesthesia, which is an important advantage over any surgical treatment (Simmons et al., 2001). More than 50 years have passed since this procedure was developed, but it has yet to become common practice. One of the main reasons for this is the low specificity for enzymes that target the NP (Brown, 1996; Nordby et al., 1996). Condoliase degrades chondroitin sulfate and hyaluronic acid chains (Takahashi, 2004). Numerous mammalian tissues contain chondroitin sulfate, including the NP, bone, and cartilage. Therefore, condoliase is a potential therapeutic enzyme for LDH (Knezevic et al., 2017; Matsuyama and Chiba, 2019). It has been verified that condoliase is significantly less harmful to the surrounding tissues, and the nervous and vascular system than chymopapain. In Japan, condoliase (HERNICORE®, Seikagaku Corporation, Tokyo, Japan) was released in 2018 for LDH treatment.

### 4.1 Effectiveness of condoliase in treating LDH

Studies show that more than 90% of LDH patients who undergo surgical treatment experience symptomatic relief within a few months (Jacobs et al., 2011). We conducted a systematic review of all available literature on the intradiscal injection of condoliase for LDH. A single-arm meta-analysis of these studies demonstrated that approximately 74%, 81%, and 79% of patients reported clinically significant pain improvement at 3, 6, and 12 months after condoliase treatment, respectively. A two-arm meta-analysis of RCT studies demonstrated that the ODI score change in the condoliase treatment group was greater than that in the placebo control group. Although there is still a gap between the efficacy of condoliase treatment and surgical treatment, approximately 78% of patients in whom conservative treatment is ineffective can achieve therapeutic effects after condoliase treatment good enough to avoid surgery. Approximately 5%–10% of patients with LDH who have undergone surgical treatment require reoperation (Sugimura et al., 1996). Our meta-analysis showed that the proportion of surgeries after condoliase treatment was 9% at the last follow-up. Therefore, chemonucleolysis with condoliase is similar to surgical treatment in preventing LDH recurrence. The pharmacological effects of condoliase involve the degradation of hyaluronic acid and dehydration of the NP, which diminishes the volume of the herniated mass; On the other hand, it may lead to further degradation of the intervertebral disc, causing signal changes in MRI and Pfirmann grading. Our two-arm meta-analysis of RCT studies demonstrated that the herniated mass volume change in the condoliase treatment group was greater than that in the placebo group. A single-arm meta-analysis showed that the proportion of patients with Pfirrmann grade change after condoliase treatment was 43% at the last follow-up. Sugimura et al. (1996); (Lønne et al., 2012) found after 28 weeks of condoliase intradiscal injection, glycosaminoglycan content recovered in monkeys, and Banno et al. (2022) found that disc degeneration caused by chemonucleolysis with condoliase could be reversed after 1 year. Thus, the effect of condoliase on the NP is only temporary, and the intervertebral disc can regenerate once enzyme activity has disappeared. The phenomenon is more prevalent in younger patients (Kobayashi et al., 2022). With the exception of the study of Banno et al. (2022), no recovery of the Pfirmann grade has been reported in any other clinical studies of condoliase; therefore, the long-term effects of condoliase require further observation.

### 4.2 Safety of condoliase in treating LDH

In an RCT study conducted by Matsuyama et al. (2018), 194 patients received condoliase intradiscal injections of 1.25, 2.5, or 5 U or a placebo intradiscal injection. The results showed that although all three doses had similar efficacy, adverse events were dose-dependent; therefore, 1.25 U of condoliase was an appropriate dose for intradiscal injections. Our meta-analysis showed that the adverse events after condoliase treatment were 4%, and the results of the two-arm meta-analysis showed no difference in adverse events between the condoliase treatment and placebo control groups. As condoliase is an exogenous protein, the risk of anaphylactic shock cannot be ignored. However, no anaphylactic shock cases were reported in the included studies. Rashes are the most common allergy-like symptoms of condoliase treatment; however, all symptoms can be resolved after standard dermatologic treatment. A small number of patients also experienced mild to moderate back pain within a week of condoliase injection, but the pain resolved or abated in most patients without treatment. No neurological deterioration or spondylitis infection was reported after condoliase intradiscal injection (Ishibashi et al., 2020; Nakajima et al., 2020; Hirai et al., 2021; Inoue et al., 2021).

### 4.3 Precautions

To prevent anaphylactic reactions, condoliase can be used only once in a lifetime, which makes it particularly important to identify likely responders and determine the indications for the use of condoliase. ①Herniation type: Sequestration herniation was not included in all studies. While some studies excluded transligamentous herniation (Chiba et al., 2018; Banno et al., 2022), others have shown that condoliase appears to be an effective treatment for all herniation types except sequestration herniation (Ishibashi et al., 2020; Nakajima et al., 2020; Hirai et al., 2021; Kobayashi et al., 2022; Oshita et al., 2022). ②Disease duration: Many reports have shown that prolonged symptom duration has an adverse impact on the prognosis of patients with LDH (Sugimura et al., 1996). Therefore, it is important to intervene at the best time rather than pursue ineffective conservative treatment. Banno et al. (2022) showed a low response rate for condoliase treatment in patients with symptoms lasting longer than 1 year. Nakajima suggested that intradiscal injection of condoliase should be performed 6 months after disease onset (Nakajima et al., 2020). ③Symptoms: The indication for condoliase intradiscal injection were symptoms of unilateral lower extremity pain with or without back pain, nerve root compression by a herniated disc confirmed using MRI, neurological signs consistent with the distribution of the compressed nerve root. LDH patients with neurological deficits such as cauda equina syndrome and severe progressive dyskinesia should not be treated with condoliase (Chiba et al., 2018; Ishibashi et al., 2018; Ishibashi et al., 2020; Nakajima et al., 2020; Hirai et al., 2021; Banno et al., 2022; Kobayashi et al., 2022). ④Age: In principle, condoliase intradiscal injection is better for young LDH patients with high water content in the NP and simple reasons for low back pain (Ishibashi et al., 2020). However, multiple clinical studies have shown that condoliase may be effective for LDH regardless of age (Hirai et al., 2021; Oshita et al., 2022). ⑤Predictive factors for better efficacy: Banno et al. (2022) and Ishibashi et al. (2020) reported that high intensity on T2-weighted MRI had a positive impact on therapeutic effects showed for chemonucleolysis. Nakajima and Ishibashi found that a larger herniated mass volume showed better efficacy for chemonucleolysis with condoliase. ⑥Risk factors: Risk factors for poor prognosis with condoliase include a history of hernia opening, presence of spondylolisthesis, or a posterior intervertebral angle of > 5° (Ishibashi et al., 2020; Hirai et al., 2021). Based on these investigations, suggestions were made for the clinical application of condoliase in treating LDH.

### 4.4 Limitations

This review and the existing literature related to condoliase have important limitations. ①This analysis only included English language studies due to language limitations. ②Though we used comprehensive search strategies in electronic databases for our study, some eligible studies may have been missed. ③Only two RCT studies on condoliase for LDH could be found, leading to only a single-arm meta-analysis for some outcomes. ④Patient characteristics such as Diabetes Mellitus, smoking, and activity levels may have an impact on the outcome of condoliase intradiscal injection, but these factors were not discussed in the included studies. ⑤Most of the included studies had short follow-up times and were inconsistent among included studies, the longer-term clinical outcomes of condoliase intradiscal injection are unknown.

## 5 Conclusion

In conclusion, our meta-analysis shows that condoliase intradiscal injection has excellent eutherapeutic and safety for LDH. Thus condoliase intradiscal injection has considerable potential as a treatment option besides conservative treatment and surgical intervention. However, the strength of this conclusion is diminished due to limitations in the quality and type of studies included in this study. In the future, large double-blinded double-arm RCT studies are still needed.

## Data Availability

The original contributions presented in the study are included in the article/Supplementary Material, further inquiries can be directed to the corresponding authors.
